# Al-Induced Unusual Grain Growth in Ni-Co-Cr Multi-Principal Element Alloys

**DOI:** 10.3390/ma19030505

**Published:** 2026-01-27

**Authors:** Kexuan Zhou, Siqi Wu, Yan Zhou, Yanjun Zhang, Xiaoxin Lei, Xin Wang, Xiaoyong Xu, Wenhao Gong, Yue Li, Zhijun Wang

**Affiliations:** 1 The First Aircraft Institute, Aviation Industry Corporation of China, Xi’an 710089, China; siqi_wu@mail.nwpu.edu.cn (S.W.);; 2 State Key Laboratory of Solidification Processing, Northwestern Polytechnical University, Xi’an 710072, China

**Keywords:** multi-principal element alloys, Ni-Co-Cr, substitutional solid solution, grain growth, apparent activation energy

## Abstract

Substitutional elements are introduced to face-centered cubic (FCC) multi-principal element alloys (MPEAs) to effectively enhance the mechanical performance by solid solution strengthening and second-phase strengthening. Commonly, relatively large atomic radius elements introduced into the alloy matrix result in lattice distortion and hinder grain boundary migration, thus achieving matrix strengthening. However, owing to the complex compositions of MPEAs, different substitutional elements introduced affect the microstructure evolution behavior and corresponding strengthening effects. In this work, an abnormal grain growth behavior of Ni-Co-Cr-based MPEAs based on Al alloying was observed. Systematic annealing experiments combined with quantitative grain growth analysis were conducted to clarify the effects of Al, W, and Mo on grain boundary migration. The results show that substitutional Al reduces the apparent activation energy for grain growth, resulting in both a lower grain growth component (*n* = 2) and a lower activation energy for grain growth of 219 kJ/mol, thereby enhancing grain boundary mobility. On the contrary, minor additions of high-melting-point W and Mo effectively inhibited the Al-induced rapid grain growth by increasing the activation energy and resulting in a higher grain growth component and a lower activation energy for grain growth of 251 kJ/mol. These findings provide new insights into the role of substitutional solutes in controlling grain growth kinetics in multi-principal element alloys.

## 1. Introduction

Ni-Co-Cr-based multi-principal element alloys have attracted considerable research interest due to their low stacking fault energy [1,2], twinning-dominated deformation mechanisms [3,4], and the presence of chemical short-range order [5,6,7]. These characteristics provide a favorable combination of strength and ductility over a wide temperature range (77 K–673 K), making this alloy system one of the most representative face-centered cubic multicomponent alloys for structural applications [2,8,9]. One effective approach to further enhance the mechanical performance of Ni-Co-Cr-based alloys is alloying [10,11,12,13,14]. The addition of suitable solute elements can introduce solid-solution strengthening and, in some cases, second-phase strengthening. Among various alloying elements, Al has been most extensively investigated. Agustianingrum et al. [13] examined the strengthening effect of Al in CoCrNi MPEAs through experimental characterization combined with theoretical analysis. Their results showed that the alloys retain a single-phase FCC solid-solution structure for Al contents up to 7 at.%, while the yield strength increases continuously with increasing Al concentration. Another important strategy for strengthening is microstructural control [15,16]. Grain refinement is widely employed, as the strength of polycrystalline materials increases with decreasing grain size according to the Hall-Petch relationship [17,18,19]. In multi-principal element alloys, grain size is typically controlled through recrystallization followed by grain growth [20,21]. Grain growth is governed by grain boundary migration, which is influenced by grain boundary chemistry, solute segregation, and the local chemical order or disorder, all of which affect grain boundary energy and mobility [22,23,24]. In addition, the near-equiatomic composition of multi-principal element alloys leads to lattice distortion and complex diffusion behavior, further complicating grain boundary migration [14,25,26,27]. Understanding grain growth in such systems is therefore essential for clarifying diffusion mechanisms, grain boundary kinetics, and microstructural thermal stability.

Currently, quantitative studies on the grain growth behavior of CoCrNi multi-principal element alloys with Al additions remain limited. Quantitative investigations of grain growth are not only of fundamental scientific interest but also of significant relevance to engineering applications. Grain size is a key microstructural parameter governing the strength and ductility of alloys. Clarifying grain growth behavior after recrystallization and establishing quantitative grain growth kinetic models enable the prediction of grain size evolution under different annealing temperatures and holding times, thereby facilitating the optimization of heat treatment processes such as solution treatment and aging. This provides a theoretical basis for microstructural control through thermal or thermomechanical processing and for achieving a balanced combination of strength and toughness.

In this work, Ni-Co-Cr-based multi-principal element alloys are systematically investigated to clarify the effects of key solute elements, including Al, W, and Mo, on grain growth behavior through quantitative experimental analysis. The results show that Al significantly accelerates grain growth. To limit the rapid grain coarsening induced by Al addition, minor amounts of W and Mo are introduced, leading to an effective suppression of grain growth. These findings highlight the important role of solute elements in controlling grain growth in multicomponent alloys and provide guidance for alloy design and microstructural control.

## 2. Materials and Methods

**Figure 1:**
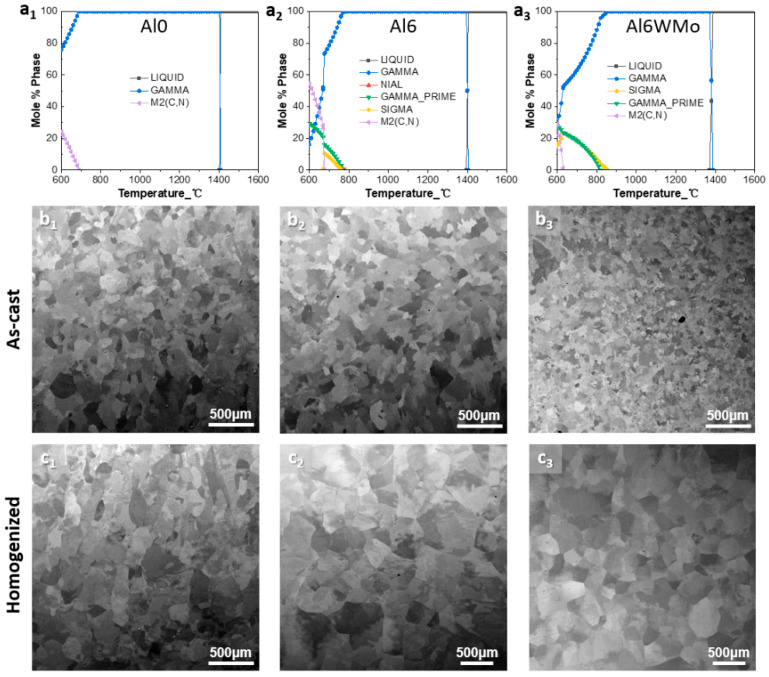
The equilibrium phase diagrams and representative BSE microstructures of the selected (**a_1_**–**c_1_**) Ni_33.34_Co_33.34_Cr_33.32_, (**a_2_**–**c_2_**) Ni_33.34_Co_33.34_Cr_27.32_Al_6_, and (**a_3_**–**c_3_**) Ni_32.34_Co_32.34_Cr_27.32_Al_6_W_1_Mo_1_ MPEAs under different states.

To quantitatively investigate the influence of Al, W, and Mo solutes on grain growth behavior of Ni-Co-Cr multi-principal element alloys, Ni_33.34_Co_33.34_Cr_33.32_, Ni_33.34_Co_33.34_Cr_27.32_Al_6_, and Ni_32.34_Co_32.34_Cr_27.32_Al_6_W_1_Mo_1_ (hereafter named Al0, Al6, and Al6WMo for short) alloys were selected. The corresponding equilibrium phase diagrams of Al0, Al6, and Al6WMo MPEAs were calculated to identify the phase transformation during the solidification, as shown in Figure 1(a_1_–a_3_), displaying that all three alloys have a single-phase FCC region over a wide temperature range. Button-shaped Al0, Al6, and Al6WMo samples were produced via vacuum arc melting using the high-purity (99.95%) raw Al, Co, Cr, Ni, W, and Mo elements in a Tigettered argon atmosphere. The obtained ingots were remelted at least five times to ensure chemical homogeneity and then poured into a water-cooled Cu mold with the dimensions of 60 mm × 12 mm × 5 mm. All the as-cast cuboid-shaped samples were subsequently homogenized and treated at 1250 °C for 2 h, followed by water quenching. Then, the homogenized samples were cold-rolled with 70% thickness reduction and directly annealed at 950 °C~1100 °C for different times, respectively, followed by water quenching. The detailed thermomechanical processing routes are summarized in Table 1.

For microstructure characterization, backscattered electron (BSE) imaging, secondary electron (SE) imaging, energy-dispersive spectrometry (EDS), and EBSD were carried out on a scanning electron microscope (SEM, TESCAN MIRA3) at 20 kV voltage. The analyzed samples of the as-cast, homogenized, and as-annealed Al0, Al6, and Al6WMo MPEAs were first cut from the bulk samples using electrical discharge machining (EDM) and then mechanically ground to approximately 50 μm using SiC paper, and finally electrolytically polished using a perchloric acid corrosive solution. Figure 1(b_1_–b_3_),(c_1_–c_3_)display the BSE microstructures of the as-cast and homogenized Al0, Al6, and Al6WMo MPEAs, indicating that all three alloys possessed a single-phase polycrystalline microstructure. The average grain size of each sample annealed at different temperatures for different times was further measured based on EBSD-characterized microstructures. To ensure the validity and accuracy of the data, EBSD characterization was conducted at different magnifications to ensure each specimen contained at least 100 grains for evaluation. EBSD testing parameters for each specimen were determined after a preliminary scan with a large step size.

## 3. Results and Discussion

**Figure 2:**
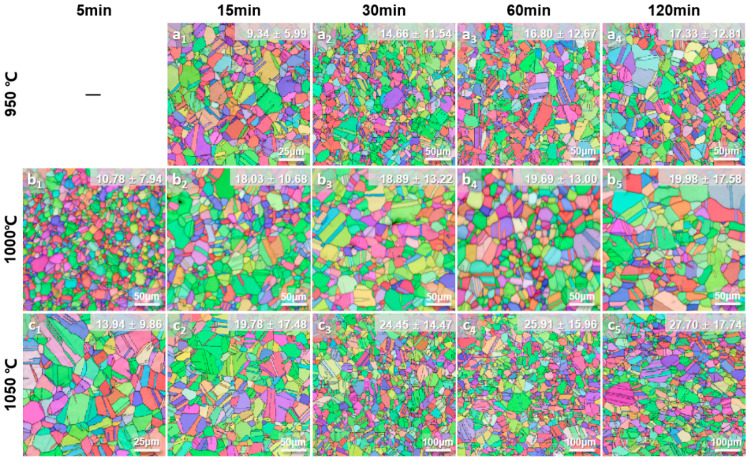
EBSD characterized microstructures in the as-annealed Al0 MPEAs heat treated at different temperatures: (**a_1_**–**a_4_**) 950 °C; (**b_1_**–**b_5_**) 1000 °C; (**c_1_**–**c_5_**) 1050 °C.

**Figure 3:**
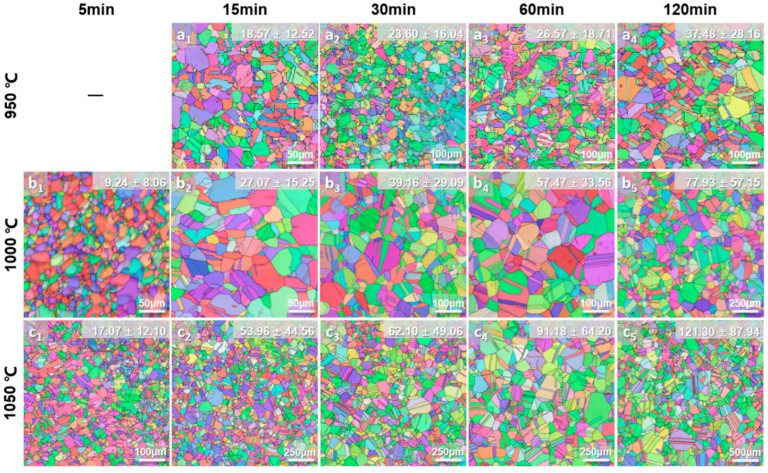
EBSD characterized microstructures in the as-annealed Al6 MPEAs heat treated at different temperatures: (**a_1_**–**a_4_**) 950 °C; (**b_1_**–**b_5_**) 1000 °C; (**c_1_**–**c_5_**) 1050 °C.

**Figure 4:**
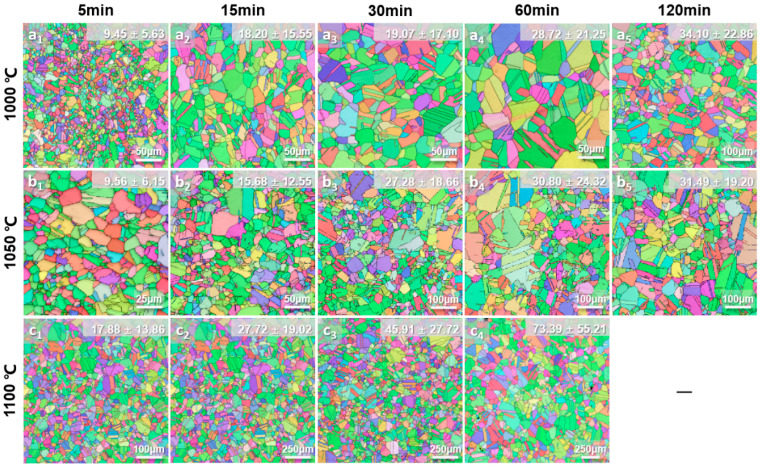
EBSD characterized microstructures in the as-annealed Al6WMo MPEAs heat treated at different temperatures: (**a_1_**–**a_5_**) 1000 °C; (**b_1_**–**b_5_**) 1050 °C; (**c_1_**–**c_4_**) 1100 °C.

Classical grain growth kinetics are commonly quantified by analyzing the grain size as a function of isothermal annealing time at different temperatures [28,29]. To systematically investigate the grain growth behavior under different substitutional solid solutions, recrystallization annealing treatments were conducted to obtain Al0, Al6, and Al6WMo MPEAs with different grain sizes. The annealed samples characterized by EBSD with different average grain sizes are shown in Figure 2, Figure 3 and Figure 4. Notably, to avoid the influence of the existence of second phases and ensure consistency in the analysis of the influence of solute elements on the grain growth behavior of Al0, Al6, and Al6WMo MPEAs, annealing temperature for each alloy was investigated based on preliminary experiments. The corresponding results are shown in Figures S1–S3 in Supplementary Information. Figure S1 shows that Al6 MPEAs annealed at 900 °C for 60 min~120 min exhibited second particles around grain boundaries, while no second phase appeared in Al0 MPEAs. To further increase the annealing temperature to 950 °C, there was no second particle forming in Al6 MPEAs. Therefore, the minimum annealing temperature for Al0 and Al6 MPEAs was chosen as 950 °C. Meanwhile, Figures S2 and S3 show that Al6WMo MPEAs annealed at 900 °C for 15 min formed second particles around grain boundaries and resulted in a non-uniform grain distribution, while there was no second phase formed after annealing at 1000 °C~1100 °C. Considering W and Mo elements with a higher melting point, the minimum annealing temperature for Al6WMo MPEAs was chosen as 1000 °C. For Al0 and Al6 alloys, the selected annealing temperatures were 950 °C, 1000 °C, and 1050 °C, which were 1000 °C, 1050 °C, and 1100 °C for the counterpart Al6WMo alloys.

It can be observed that all samples underwent complete recrystallization and exhibited equiaxed microstructures containing a high density of annealing twins. Further statistical analysis revealed that after annealing at 950 °C~1050 °C for 5~120 min, the fraction of twin boundaries in Al0 MPEAs remains ~56%. Meanwhile, the corresponding twin boundary fraction in the Al6 MPEAs is about 53.5%, which is slightly lower than that of the former. However, a notable difference is observed in the twin boundary fraction of the Al6WMo alloy, which shows a slight dependence on annealing temperature. Specifically, the twin boundary fraction is approximately 52.5% at annealing temperatures of 1000 °C and 1050 °C, whereas it increases to about 59% when the annealing temperature is raised to 1100 °C. At higher annealing temperatures, the co-doping of Al, W, and Mo promotes the formation of annealing twins in the Ni-Co-Cr-based multicomponent alloy matrix. A similar phenomenon has also been reported by Zhang et al. [30].

The average grain sizes of Al0, Al6, and Al6WMo MPEAs were measured using an AztecCrystal software (Version:2.1, Make: Oxford Instrument plc, Abingdon, Oxfordshire, UK), as indicated in the upper-right corner of each figure and listed in Table 2. The average grain size of all three MPEAs increased progressively with the increasing annealing temperature and holding time. The grain size of Al6 MPEAs showed a significantly higher grain growth rate over time compared to the Al0 and Al6WMo MPEAs. In particular, after annealing at 1050 °C for 120 min, the average grain size of Al6 MPEA reached approximately 121 μm, which was substantially larger than that of the Al0 and Al6WMo counterparts under the same heat treatment condition (~28 μm and 31 μm), demonstrating that Al replaces the same content of Cr, accelerating grain growth in Ni-Co-Cr-based MPEAs, while minor additions of W and Mo effectively suppress the rapid grain growth induced by Al substitutional solid solution instead.

**Figure 5:**
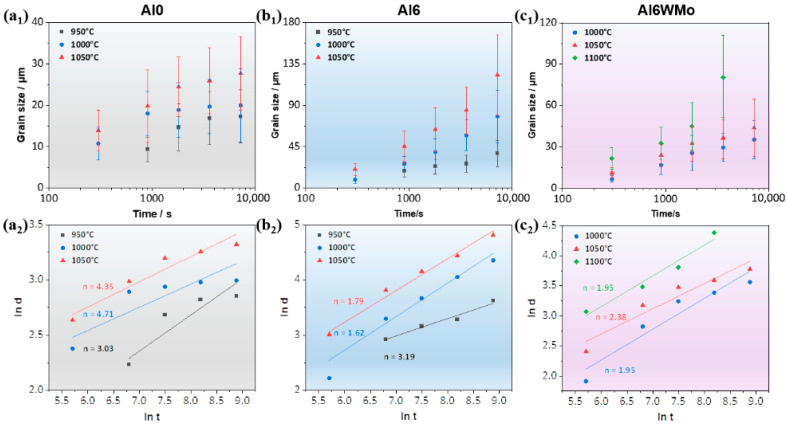
The grain sizes of Al0, Al6, and Al6WMo MPEAs changed with annealing time during annealing treatments: The (**a_1_**) linear and (**a_2_**) double logarithmic plots of the average grain size with annealing time in the Al0 alloy. The (**b_1_**) linear and (**b_2_**) double logarithmic plots of the average grain size with annealing time in the Al6 alloy. The (**c_1_**) linear and (**c_2_**) double logarithmic plots of the average grain size with annealing time in the Al6WMo alloy.

Figure 5(a_1_–c_1_) show the evolution of recrystallized grain size as a function of annealing time for the Al0, Al6, and Al6WMo MPEAs during annealing in the temperature range of 950 °C–1100 °C. It is observed that the average grain size of the Al0 alloy gradually approaches a steady value with increasing annealing time, whereas the average grain size of the Al6 alloy increases linearly with annealing time. By comparison, the increase in average grain size of the Al6WMo alloy is slower than that of the Al6 alloy. Based on empirical analysis, Grey and Higgins proposed that grain growth slows down as the average grain size increases, and that grain growth eventually ceases when the driving force for grain boundary migration, p, decreases to a critical value. This behavior may originate from the presence of slowly diffusing solute atom clusters in solid solutions, which act in a manner similar to second-phase particles and exert a pinning effect on grain boundaries. To further quantify the effects of Al, W, and Mo on grain growth in Ni-Co-Cr-based FCC multicomponent alloys, the grain growth kinetics were evaluated using the model proposed by Burke and Turnbull, as expressed in Equation (1) [28]:

(1) $$d^{n}-d_{0}^{n}=kt$$

where d is the instantaneous grain size at a given annealing time, $d_{0}$ is the initial grain size, n is the grain growth exponent, k is the grain growth kinetic constant at a given temperature, and t is the annealing time. For cold-rolled alloys subjected to large plastic deformation, the initial grain size can be reasonably assumed to be zero [21,31]. Accordingly, Equation (1) can be simplified to $d^{n}=kt$. The grain growth exponent at different temperatures can then be obtained by fitting the double-logarithmic plots of average grain size versus annealing time. Figure 5(a_2_–c_2_) further present the double-logarithmic plots of average grain size versus annealing time for the Al0, Al6, and Al6WMo alloys at different annealing temperatures, together with the corresponding fitted grain growth exponents n. It can be seen that the n values of all three alloys fall within the range of 2–4, among which the Al0 alloy generally exhibits higher n values.

Based on the determined grain growth exponents of the Al0, Al6, and Al6WMo alloys, the corresponding grain growth activation energies can be further evaluated. The grain growth kinetic constant k follows an Arrhenius-type relationship with temperature, as given by Equation (2):

(2) $$k=k_{0}exp\left(-\frac{Q}{RT}\right)$$

where $k_{0}$ is a constant, Q is the grain growth activation energy (kJ/mol), R is the gas constant (8.3145 J/(mol∙K)), and T is the annealing temperature (K). Combining Equations (1) and (2) yields $d^{n}/t=k_{0}exp(-Q/RT)$. Taking the natural logarithm of both sides gives the following:

(3) $$\ln\left(\frac{d^{n}}{t}\right)=lnk_{0}-\frac{Q}{RT}$$

**Figure 6:**
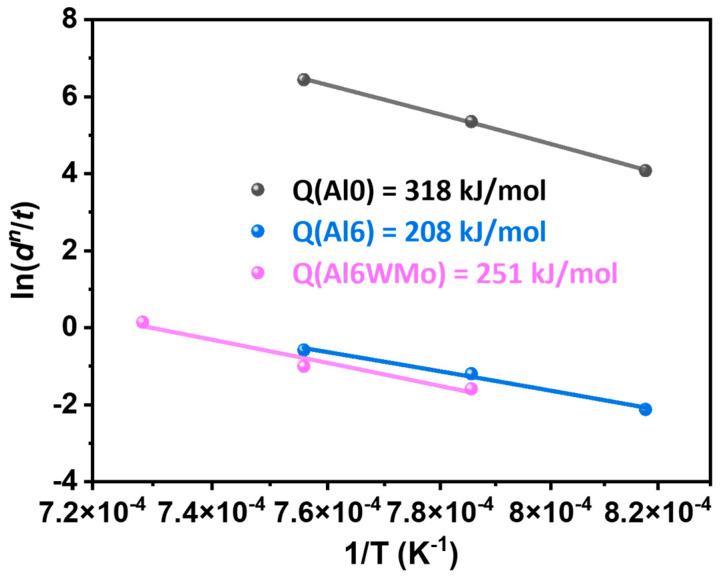
The grain growth activation energy of the Al0, Al6, and Al6WMo MPEAs.

Accordingly, $ln(d^{n}/t)\;$exhibits a linear dependence on 1/T, with a slope equal to Q/R. Based on this relationship, the grain growth activation energies of the Al0, Al6, and Al6WMo multicomponent alloys are determined to be 318 kJ/mol, 208 kJ/mol, and 251 kJ/mol, respectively, as shown in Figure 6. A larger activation energy indicates a greater resistance to grain growth [31,32].

Grain growth is a process involving a reduction in the total number of grains and an increase in the average grain size, which is governed by the migration of high-angle grain boundaries (HAGBs). The relationship between the grain boundary migration velocity v and the driving pressure p can be described by Equation (4) [28]:

(4) $$v=Mp=\frac{b^{2}D_{d}}{k_{B}T}p$$

where M is the grain boundary mobility, b is the grain boundary spacing, $D_{d}$ is the grain boundary diffusion coefficient, $k_{B}$ is the Boltzmann constant, and p is the driving pressure arising from interfacial curvature. Grain boundary mobility depends on temperature and crystallographic factors, such as grain boundary misorientation and boundary character, and is also strongly influenced by impurity concentration. Even trace amounts of solute can significantly affect grain boundary mobility and, in some cases, reduce it by several orders of magnitude. In most cases, when the impurity concentration is low, the apparent activation energy for grain boundary migration is comparable to that for self-diffusion or for solute diffusion in the solvent [28].

The effects of different alloying elements on grain boundary mobility, therefore, depend on their self-diffusion activation energies. The self-diffusion activation energy $Q_{s}$ is the sum of the migration activation enthalpy and the vacancy formation energy, both of which are associated with the breaking of bonds between neighboring atoms [14]. The melting point of an alloy can be approximately regarded as the temperature required to overcome all bonding energies and is thus related to bond strength. For conventional FCC pure metals, the diffusion activation energy approximately follows the Van Liempt relationship with the melting point $T_{m}\;$[33], given by $Q_{s}\approx 0.1422T_{m}\;$(kJ/mol). Accordingly, metallic elements with higher melting points generally exhibit stronger interatomic bonding and higher self-diffusion activation energies [33,34].

Based on the experimentally observed grain growth behavior of the single-phase FCC Al0, Al6, and Al6WMo multicomponent alloys, it can be concluded that substituting the low-melting-point element Al ($T_{m}=660$ °C) for an equivalent amount of the high-melting-point element Cr ($T_{m}=1857$ °C) reduces the apparent activation energy for grain boundary migration. This reduction enhances grain boundary mobility and thereby accelerates grain growth. In contrast, the introduction of higher-melting-point elements W ($T_{m}=3407$ °C) and Mo ($T_{m}=2610$ °C) leads to the opposite effect. Chang et al. [35] reported slow recrystallization kinetics in NiCoCr multicomponent alloys with W additions and attributed this behavior to the large atomic radius of W relative to the matrix elements, which induces lattice distortion and impedes dislocation motion and grain boundary migration during recrystallization. However, in the present study, Al—despite having a larger atomic radius than W and Mo—promotes grain growth. This observation indicates that, compared with atomic size, the self-diffusion activation energy of solute elements plays a more dominant role in governing grain growth behavior in multicomponent alloys [36]. It is also worth noting that the recrystallization microstructural evolution of the Al-substituted Ni-Co-Cr-based FCC multicomponent alloys examined in this work was conducted at relatively high annealing temperatures, thereby avoiding an increase in grain growth activation energy caused by precipitate pinning effects [29,31].

## 4. Conclusions

In summary, Al and W, as well as Mo elements with a larger atomic radius but different melting points, were selected as alloying solutes to quantitatively study their effects on the grain growth behavior of the single-phase Ni-Co-Cr FCC MPEAs. All specimens were produced by arc melting, copper mold casting, and then thermomechanical processing to obtain fully recrystallized single-phase FCC MPEAs with different grain sizes. Based on the EBSD results, it can be found that substituting Cr with an equivalent amount of Al element, which has a low melting point and low elastic modulus, significantly promoted grain growth and resulted in both a lower grain growth component (*n* = 2) and a lower activation energy for grain growth of 219 kJ/mol. On the contrary, minor W and Mo solutes further substituting Ni and Co elements in Al6 MPEAs produce a solute-drag effect to some extent and result in a higher grain growth component and a lower activation energy for grain growth of 251 kJ/mol. All three elements have a larger atomic radius compared to the Ni-Co-Cr MPEA matrix, yet they display different grain growth behavior, indicating that the self-diffusion activation energy of solute elements has a more significant effect on the grain growth behavior of multi-principal element alloys than the atomic size. This work provides a more comprehensive perspective on the influence of solid solutes on grain boundary migration in single-phase Ni-Co-Cr-based multi-principal element alloys.

## Figures and Tables

**Table 1:** Thermomechanical processes used in the as-cast Al0, Al6, and Al6WMo MPEAs.

Alloy	Name	Solid Solution Treatment	Cold Rolling	Recrystallization/Aging Treatment
Ni_33.34_Co_33.34_Cr_33.32_	Al0	1250 °C/2 h	70%	1050 °C/5~120 min
				1000 °C/5~120 min
				950 °C/5~120 min
Ni_33.34_Co_33.34_Cr_27.32_Al_6_	Al6	1250 °C/2 h	70%	1050 °C/5~120 min
				1000 °C/5~120 min
				950 °C/5~120 min
Ni_32.34_Co_32.34_Cr_27.32_Al_6_W_1_Mo_1_	Al6WMo	1250 °C/2 h	70%	1100 °C/5~120 min
				1050 °C/5~120 min
				1000 °C/5~120 min

**Table 2:** The average grain size of annealed Al0, Al6, and Al6WMo MPEAs was measured based on EBSD characterization.

Temperature/°C	Duration/Min	Grain Size/μm		
		Al0	Al6	Al6WMo
950	15	9.34 ± 5.99	18.57 ± 12.52	—
	30	14.66 ± 11.54	23.60 ± 16.04	—
	60	16.80 ± 12.67	26.57 ± 18.71	—
	120	17.33 ± 12.81	37.48 ± 28.16	—
1000	5	10.78 ± 7.94	9.24 ± 8.06	9.45 ± 5.63
	15	18.03 ± 10.68	27.07 ± 15.25	18.20 ± 15.55
	30	18.89 ± 13.22	39.16 ± 29.09	19.07 ± 17.10
	60	19.69 ± 13.00	57.47 ± 33.56	28.72 ± 21.25
	120	19.98 ± 17.58	77.93 ± 57.15	34.10 ± 22.86
1050	5	13.94 ± 9.86	20.36 ± 13.29	9.56 ± 6.15
	15	19.78 ± 17.48	45.20 ± 34.99	15.68 ± 12.55
	30	24.45 ± 14.47	63.64 ± 47.57	27.28 ± 18.66
	60	25.91 ± 15.96	84.54 ± 51.37	30.80 ± 24.32
	120	27.70 ± 17.74	122.90 ± 86.91	31.49 ± 19.20
1100	5	—	—	17.88 ± 13.86
	15	—	—	27.72 ± 19.02
	30	—	—	45.91 ± 27.72
	60	—	—	73.39 ± 55.21

## Data Availability

The original contributions presented in this study are included in the article/Supplementary Materials. Further inquiries can be directed to the corresponding authors.

## Supplementary Materials

The following supporting information can be downloaded at: https://www.mdpi.com/article/10.3390/ma19030505/s1.
