# Supplementary material for: Al-Induced Unusual Grain Growth in Ni-Co-Cr Multi-Principal Element Alloys
Source: Materials (Basel). 2026 Jan 27;19(3):505. doi: 10.3390/ma19030505 (PMC12897969; doi:10.3390/ma19030505)
Supplement: Supplementary file 1 [file materials-19-00505-s001.zip › materials-4093334-supplementary.pdf]

Supplementary materials

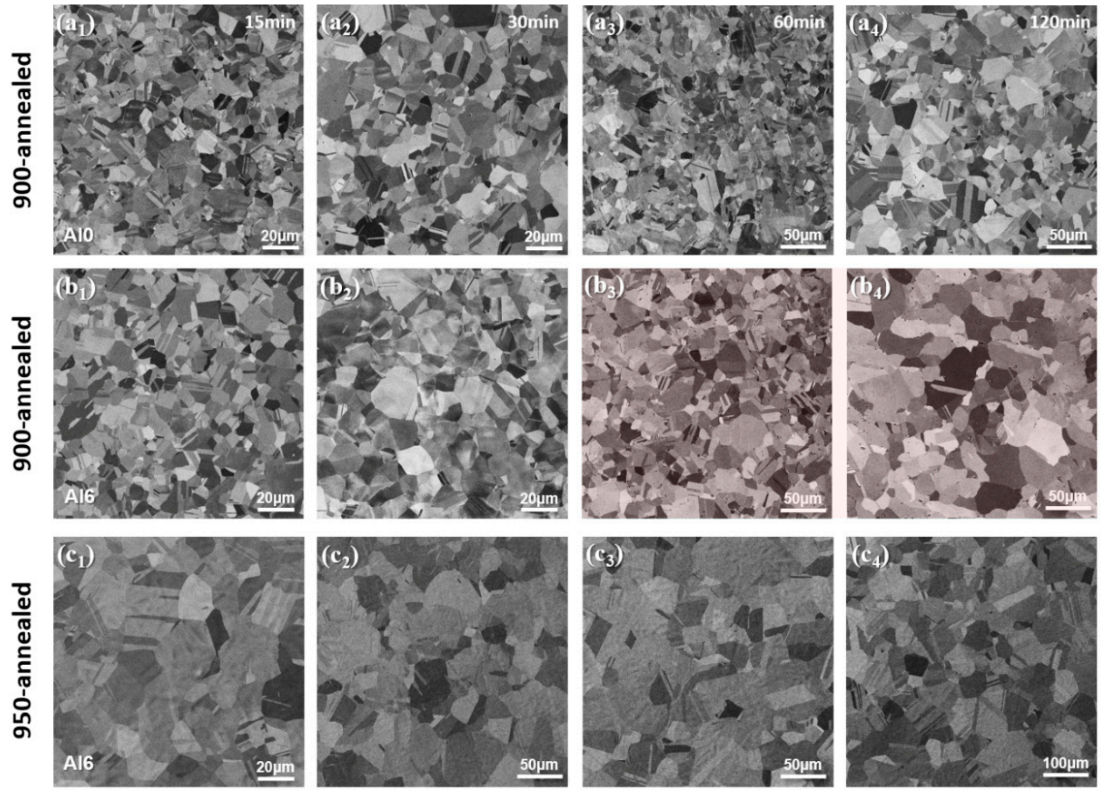

Figure S1 BSE characterized microstructures of the $Ni_{33.34}Co_{33.34}Cr_{33.32-x}Al_x$ (x = 0, 6) MPEAs annealed at 900 ℃ and 950 ℃ for different time: (a_1-a_4) Al0 ; (b_1-b_4) Al6; (c_1-c_4) Al6

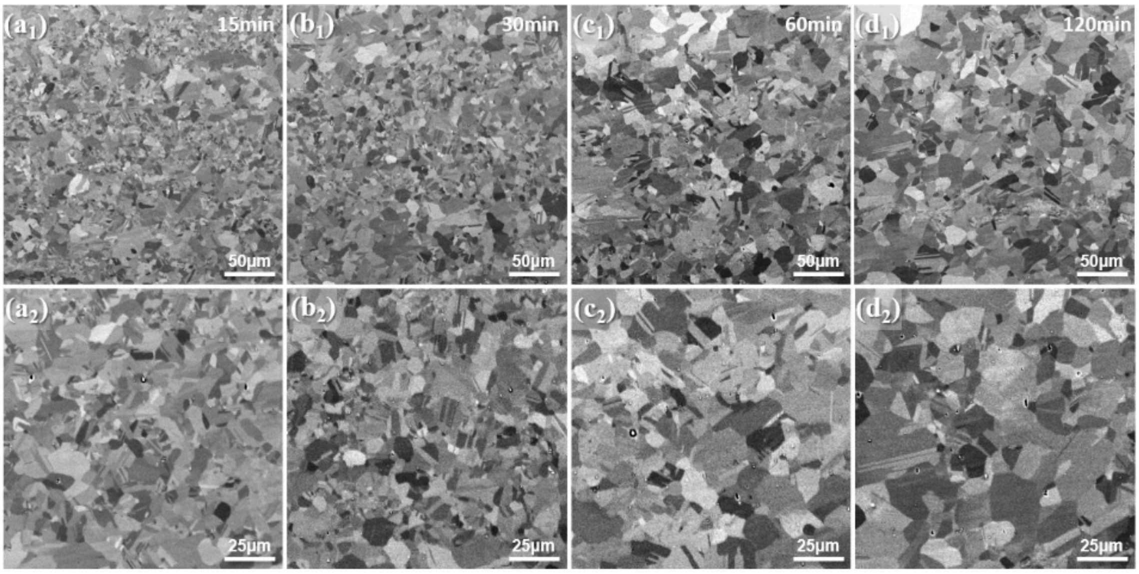

Figure S2 BSE characterized microstructures of the Al6WMo MPEAs annealed at 900 ℃ for different time: (a_1, a_2) 15 min; (b_1, b_2) 30 min; (c_1, c_2) 60 min; (d_1, d_2) 120 min

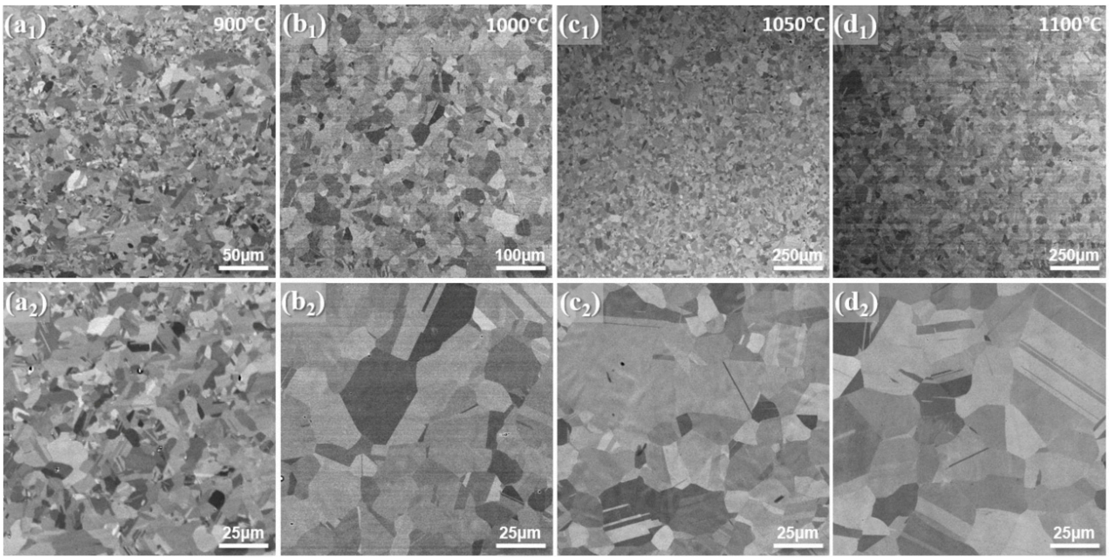

Figure S3 BSE characterized microstructures of theAl6WMo MPEAs annealed at different temperatures for 15 min: (a_1, a_2) 900 ℃; (b_1, b_2) 1000 ℃; (c_1, c_2) 1050 ℃; (d_1, d_2) 1100 ℃
